# Pretreatment of South African sugarcane bagasse using a low-cost protic ionic liquid: a comparison of whole, depithed, fibrous and pith bagasse fractions

**DOI:** 10.1186/s13068-018-1247-0

**Published:** 2018-09-11

**Authors:** Clementine L. Chambon, Thandeka Y. Mkhize, Prashant Reddy, Agnieszka Brandt-Talbot, Nirmala Deenadayalu, Paul S. Fennell, Jason P. Hallett

**Affiliations:** 1 Department of Chemical Engineering, Imperial College London, Exhibition Road, London, SW7 2AZ UK; 2 Department of Chemistry, Durban University of Technology, P.O. Box 1334, Durban, 4000 South Africa

**Keywords:** Sugarcane bagasse, Depithing, Ionic liquid, Pretreatment

## Abstract

**Background:**

Sugarcane bagasse is an abundant and geographically widespread agro-industrial residue with high carbohydrate content, making it a strong candidate feedstock for the bio-based economy. This study examines the use of the low-cost protic ionic liquid triethylammonium hydrogen sulfate ([TEA][HSO_4_]) to fractionate a range of South African sugarcane bagasse preparations into a cellulose-rich pulp and lignin. The study seeks to optimize pretreatment conditions and examine the necessity of applying a depithing step on bagasse prior to pretreatment.

**Results:**

Pretreatment of five bagasse preparations, namely whole, industrially depithed, laboratory depithed (short and long fiber) and pith bagasse with [TEA][HSO_4_]:[H_2_O] (4:1 w/w) solutions produced highly digestible cellulose-rich pulps, as assessed by residual lignin analysis and enzymatic hydrolysis. Pretreatment under the optimized condition of 120 °C for 4 h produced a pretreated cellulose pulp with up to 90% of the lignin removed and enabled the release of up to 69% glucose contained in the bagasse via enzymatic hydrolysis. Glucose yields from whole and depithed bagasse preparations were very similar. Significant differences in lignin recovery were obtained for laboratory depithed bagasse compared with whole and industrially depithed bagasse. The silica-rich ash components of bagasse were seen to partition mainly with the pulp, from where they could be easily recovered in the post-hydrolysis solids.

**Conclusions:**

The five bagasse preparations were compared but did not show substantial differences in composition or cellulose digestibility after pretreatment. Evidence was presented that a depithing step appears to be unnecessary prior to ionoSolv fractionation, potentially affording significant cost and energy savings. Instead, lignin re-deposition onto the pulp surface (and, in turn, particle size and shape) appeared to be major factors affecting the conditioning of bagasse with the applied IL. We show that pith bagasse, a common by-product of paper making, can be successfully conditioned for high glucose release while allowing recovery of lignin and silica-rich ash. The glucose yields obtained for bagasse using [TEA][HSO_4_]-water mixtures were ~ 75% as high as for conventional aprotic ionic liquids such as [Emim][OAc]; this result is highly promising for commercialization of ionoSolv processing given [TEA][HSO_4_] is 40 times less expensive, thermally stable and recyclable.

**Electronic supplementary material:**

The online version of this article (10.1186/s13068-018-1247-0) contains supplementary material, which is available to authorized users.

## Background

Sugarcane (*Saccharum officinarum*) is a major crop in South America, Asia and Africa, containing sucrose stored in the stalk [1]. The sucrose is a commodity food supply and is traded worldwide in a refined form. The sugar is also increasingly used a feedstock for first generation bioethanol and affords the highest carbon dioxide savings of any first generation biofuel feedstock [2, 3]. Sugarcane bagasse is the fibrous lignocellulosic residue remaining after sugarcane juice extraction. As a by-product of one of the largest agro-industrial processes in the world, it is estimated that over 500 million wet tons (~ 250 million dry tons) of sugarcane bagasse are produced annually [4]. With currently available boiler technology, about half of the produced bagasse is incinerated to meet the energetic requirements of the sugar and ethanol mills; the remainder is stockpiled [5]. Valorization of the surplus bagasse in addition to the sugarcane juice has the potential to double bioethanol production per hectare without expanding cane fields or jeopardizing food and feed production [6]. Bagasse could also be used to produce other value-added products such as glucose-derived molecules and plastics [7, 8] or lignin-derived chemicals and fuels [9, 10] in order to diversify the product portfolio of the sugarcane industry, making it more resilient and competitive.

Sugarcane bagasse presents a great morphological heterogeneity in terms of its particle size and fiber composition [11]. Whole bagasse contains up to 65 wt% fiber, which is currently the most useful component as it can be used for pulp and paper production [12]. Pith, a non-fibrous tissue with higher ash content and poorer morphology, makes up ~ 30 wt% of bagasse and is problematic for paper manufacture owing to its hygroscopicity and low density [11, 13, 14]. Though the fiber and pith have almost the same chemical composition [13], their anatomical structures are quite different: pith cells are spongy particles of 0.14 × 0.5 mm length, while fiber structures have reported dimensions of 0.02 × 1.2 mm on average [15, 16]. Generally, pith particles have smaller aspect ratios (< 3) than fiber particles (> 3) and consequently have high specific surface area [17], though it should be noted that a wide range of particle shapes is expected in any sample of bagasse [16]. Bagasse also contains a moderate amount of silica [18] and other inorganics (i.e., ash) which are concentrated in the pith portion (up to 5 wt%) [13], which can lead to abrasion and damage to grinding equipment [19, 20].

Removal of the pith from the useful fiber portion has long been considered desirable to improve process efficiency and paper quality during commercial pulp and paper-making [13, 15, 21] and to reduce dust formation and spontaneous combustion hazards during bagasse stockpiling [17, 22]. This is because the higher hygroscopic ability of pith leads to increased chemicals consumption and filtration and drainage difficulties that slow down the pulping process [15, 23]. To this end, industrial depithing is used to increase the fiber content of bagasse from 60 to 80 wt% before paper pulping [13, 17]. Commercial depithing operations consist of dry, moist and wet processes wherein bagasse is mechanically abraded to break the clusters of pith off from the exploitable fibrous portion, followed by screening [5]. However, depithing entails high capital costs and energy requirements and has the potential to damage fibers, resulting in loss of material which could otherwise be used for ethanol production [17, 24]. Very limited work in the literature has examined the effect and necessity of depithing on bagasse conditioning prior to pretreatment and enzymatic hydrolysis [25, 26]. In the most representative study, Hernández-Salas et al. [26] compared the effect of dilute acid pretreatment on depithed bagasse and on pith itself. No significant differences were found in the enzymatic hydrolysis yield of pretreated materials, nor in the ethanol yields following fermentation of the resulting hydrolyzates. Avoiding the depithing step could lead to significant cost and energy savings [5, 24], making bagasse even more attractive as a feedstock.

The use of lignocellulosic biomass such as bagasse as an industrial feedstock can reduce greenhouse gas emissions and displace fossil feedstocks in the production of energy, materials, chemicals and transportation fuels [27–29]. While producing chemicals and transportation fuels from sucrose is well established, utilizing lignocellulose is currently limited by high processing costs and technological constraints, notably in the pretreatment step. This is due to the inherent inefficiency of extracting lignin, a highly recalcitrant cell wall polymer, without significant loss of carbohydrates [30]. The selection of ecologically acceptable, low-cost and abundant feedstocks as well as energy-efficient and economically viable biorefinery processes are critical bottlenecks in the development of large-scale biofuels and renewable chemicals production [31].

Pretreatment technologies for bagasse need to be able to handle its high moisture content (~ 50 wt%) [11], moderate ash content leading to abrasion of comminution equipment [1, 13] and inhomogeneous fiber morphology [11]. A variety of methods have been applied for pretreatment of lignocellulosic biomass, but only a handful show promise for large-scale commercial application [32–34]. Ionic liquids (ILs) are low-melting organic salts, some of which have potential to be used as industrial solvents for lignocellulose pretreatment. In comparison to organic solvents, they exhibit advantageous properties including non-flammability and low vapor pressure [7]. ILs may be classified as either aprotic or protic, wherein the protic subset is easily produced by combining a Brønsted acid with a Brønsted base, a single-step process involving only proton transfer [35]. Historically, however, the most intensely studied ILs were cellulose-dissolving ILs which were predominantly aprotic (APILs), requiring a large number of synthesis steps, resulting in high bulk cost. Among these, 1-ethyl-3-methylimidazolium acetate ([Emim][OAc]) results in high sugar release from virtually any feedstock type [36], but is thermally unstable at typical pretreatment temperatures [37] and suffers from reduced performance in the presence of water [38]. Hou et al. [39] demonstrated the use of “bionic liquid” cholinium lysinate ([Ch][Lys]) in the presence of 50 wt% water, displaying excellent pretreatment efficiency with > 80% glucose yields after pretreatment at 90 °C for 6 h, though biomass loadings were limited to 5 wt%. Further, the amino acid APIL used is thermally unstable and likely to be expensive to synthesize, limiting its commercial applications [40]. These drawbacks have motivated research into the development of moisture-tolerant, easily synthesized and inexpensive protic ionic liquids (PILs) with comparable performance [41–43]. Recently, Rocha et al. [44] achieved 68% glucose yields following pretreatment of sugarcane bagasse at 10 wt% loading using PIL [H_3_N(CH_2_)_2_OH][OAc]:[H_2_O] (5:1 w/w) at 150 °C for 3.5 h, though a severe (~ 33%) reduction in hydrolysis yields was observed after five successive recycles of the PIL. Deep eutectic solvents, for instance urea–choline chloride mixtures, have also been explored as less costly alternatives to APILs [45–47], with glucose yields of 60–95% reported for various agricultural residues including rice straw [48], wheat straw [49] and corncob [46]. However, these mixtures also suffer from low thermal stability [50] and their ability to be reused and recycled in a viable process remains to be demonstrated [49].

The recently developed ionoSolv pretreatment uses low-cost [51], thermally stable and recyclable [38] PILs that could be manufactured at bulk scale for as little as $1.24/kg. This puts them at price parity with common organic solvents [38, 51], far cheaper than the reference APIL [Emim][OAc], which has a bulk cost estimated at over $50/kg [38, 52]. Hallett et al. [51] have designed [HSO_4_]-based PILs with poor nucleophilic character, linked to greater thermal stability, and combined them with inexpensive alkylamines. These PILs are, therefore, suitable for large-scale use for biomass processing at elevated temperatures [32]. Previous studies by our group have demonstrated the use of PIL triethylammonium hydrogen sulfate ([TEA][HSO_4_]) to selectively extract lignin and hemicelluloses from the grass *Miscanthus* [32, 53] and the hardwood willow [54], producing a cellulose-rich pulp with greatly enhanced hydrolysis yields. A separately recovered lignin has been shown to be depolymerized, with very low ash and sulfur content, thereby contributing an additional stream with potential value as a precursor for the production of renewable aromatic chemicals [55]. [HSO_4_]-anion-based ILs have been shown to be significantly more stable than [Emim][OAc] and could be successfully recycled (by distillation to regenerate a concentrated IL solution) and reused at least four times, as demonstrated in previous studies [32, 38]. [TEA][HSO_4_] could, therefore, be an ideal candidate solvent for high-volume economically effective conversion of sugarcane bagasse.

The aim of the present study was to examine the delignification and enzymatic hydrolysis of whole and depithed South African sugarcane bagasse using a low-cost, thermally stable and recyclable IL-water solution. We also set out to investigate the effect of depithing on the performance of ionoSolv pretreatment. We used the ionic liquid [TEA][HSO_4_] to pretreat washed and air-dried bagasse to be as close to an industrially relevant scenario as possible, as storage of dried residues for processing later in the season is common in the sugarcane industry. The influence of a significant amount of pith present in the feedstock is discussed and pretreatment efficacy is gauged by analysis of the pulp lignin content and enzymatic hydrolysis of the pretreated pulp.

## Methods

### Materials

All chemicals used were of reagent grade from Sigma-Aldrich and used as received, unless otherwise noted. Whole sugarcane bagasse (*Saccharum officinarum*) (WB) was sourced from a local pulp and paper mill in the Kwazulu-Natal province of South Africa. Depithed bagasse (DB) was obtained from the same mill, where the biomass had been subjected to an industrial moist depithing process, a two-stage mechanical process consisting of hammer milling of moist bagasse (~ 50 wt% moisture) followed by screening (8 mm aperture) to remove large quantities of pith, short fibers and undesirable particulates (e.g., sand) and leave behind industrially depithed bagasse. The feedstocks were received with ~ 50 wt% moisture and were washed with deionized water to remove adhering inorganic debris and air dried at room temperature prior to shipping to the laboratory. Both fractions were then further ground with a cutting mill (sieve size 1 mm) and sieved (0.18–0.85 mm, US mesh scale − 20/+ 80) and stored air dry in sealed plastic bags at ambient temperature.

### Laboratory depithing

**Fig. 1:**
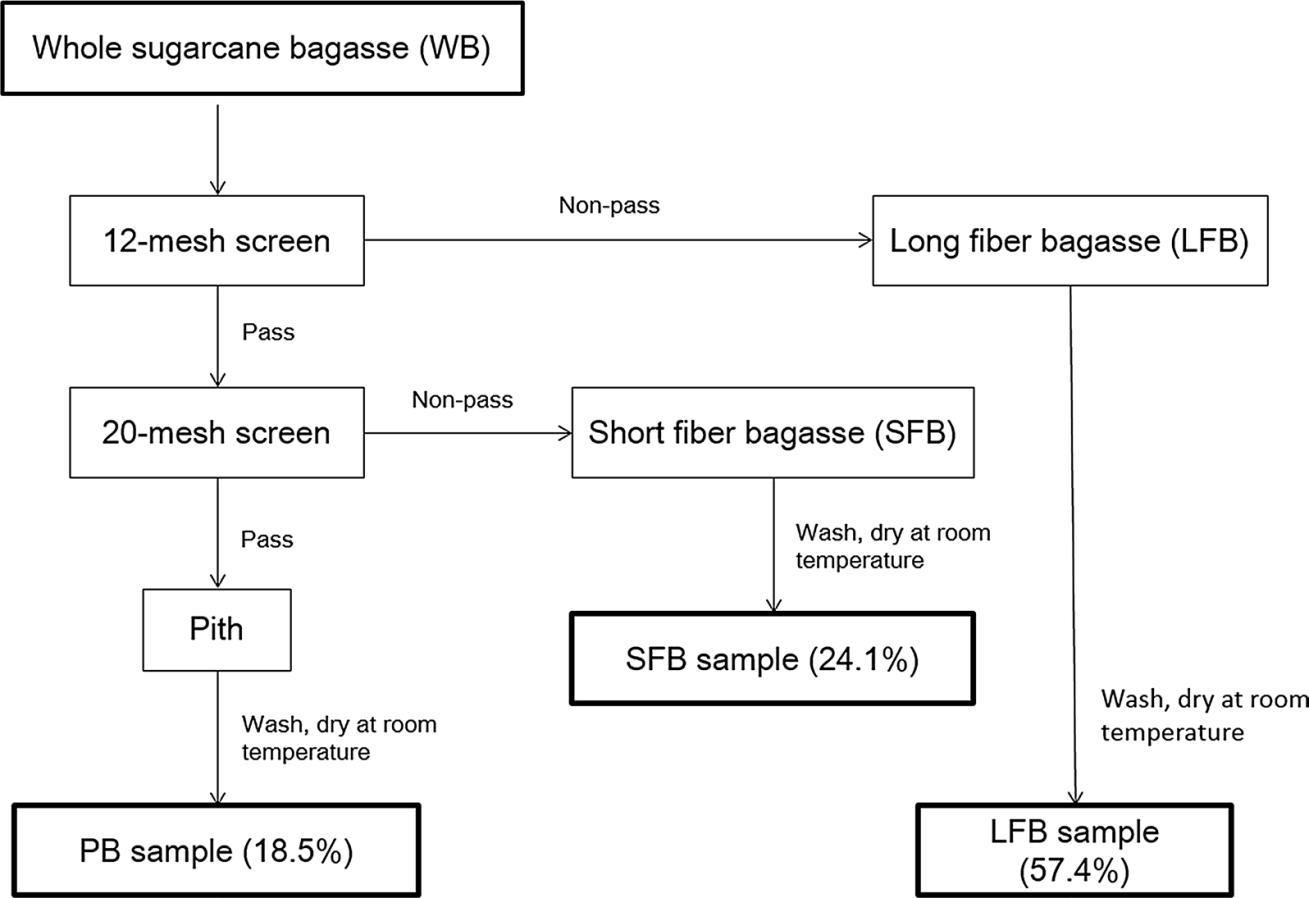
Schematic representation of laboratory depithing process

Washed whole bagasse obtained with 50 wt% moisture content from the same mill as above (but during a different harvesting period) was subjected to a laboratory fractionation process (Fig. 1) using an automated pith–fiber separator, developed and described by Chinsamy et al. [56]. The apparatus uses water and air for agitation of bagasse and separation through two screening stages of 1.6 mm (12-mesh) and 0.85 mm (20-mesh) diameter, respectively. The bagasse preparations obtained in order of decreasing particle size were long fiber bagasse (LFB), short fiber bagasse (SFB) and pith bagasse (PB). Each of the three fractions was further ground with a cutting mill (sieve size 1 mm) and sieved (0.18–0.85 mm, US mesh scale − 20/+ 80) and stored air dry in sealed plastic bags at ambient temperature.

### Ionic liquid synthesis

Triethylammonium hydrogen sulfate [TEA][HSO_4_] was synthesized as described previously [32]. Briefly, triethylamine (126.49 g, 1.25 mol) was added dropwise to a solution of 5 M H_2_SO_4_ (250 mL, 1.25 mol) in an ice bath under stirring. Water was removed using a rotary evaporator at 40 °C to reduce water content to 20 wt%. This mixture of TEA][HSO_4_]:H_2_O (4:1 w/w) was used for all pretreatment experiments. The water content was verified by Karl-Fischer titration (Mettler Toledo V20) in triplicate. The acid–base ratio (1:1) was verified by determining the density of 4:1 w/w [TEA][HSO_4_]:H_2_O solution (Anton Paar DMA 38 density meter; accuracy ± 0.0001 g cm^−3^) as 1.1910 ± 0.0004 g cm^−3^, according to a method described previously [57].

### Ionic liquid pretreatment

Ionic liquid fractionation was carried out according to a standard operating procedure of our laboratory [58]. A biomass to solvent ratio of 1:10 g/g (i.e., 10 wt% biomass loading) was used with [TEA][HSO_4_]:H_2_O (4:1 w/w) solution as the solvent. Briefly, pretreatment was carried out in triplicates at 120 °C for a residence time ranging between 1 and 24 h. After pretreatment, the pulp-IL slurry was subjected to four ethanol washes followed by an ethanol Soxhlet extraction step. The pulps were recovered by air-drying and weighed, then the moisture content was determined using NREL/TP-510-42618 [59]. After ethanol removal by evaporation, water was added to the concentrated IL to precipitate lignin, which was separated by centrifugation and recovered by drying in a vacuum-oven. A detailed description of the methods, including calculation of the pulp and lignin yields, is given in Additional file 1.

### Enzymatic hydrolysis assay

Hydrolysis assays were carried out on air-dried untreated and pretreated substrates in triplicate with blanks (also in triplicate). The assay was adapted from NREL/TP-510-42629 [60]. All substrates were hydrolyzed using a 1% (w/w) solid loading using NS22201 enzymes (Novozymes, USA). Enzymatic hydrolysis proceeded for 7 days at 50 °C and, at the end, the samples were filtered and the liquids analyzed by HPLC (Shimadzu, Aminex HPX-87P from Bio-Rad, 300 × 7.8 mm) for soluble sugars (glucose and xylose). Glucose and xylose yields were calculated as a percentage of the glucan and xylan content of untreated biomass feedstock as determined by compositional analysis. Full methods and calculation of glucose and xylose yields are described in Additional file 1.

### Compositional and Klason analysis

The composition of the five untreated bagasse samples was determined according to the protocol NREL/TP-510-42618 [59]. Prior to undertaking compositional analysis, the extractives were removed from untreated biomass following NREL-TP-510-42619 [61]. Moisture contents of untreated materials were determined following NREL/TP-510-42618 [59] as 6.8% (WB), 5.2% (DB), 8.2% (PB), 6.6% (SFB) and 6.7% (LFB). Compositional analysis was conducted in triplicate on untreated extracted biomass feedstocks. Briefly, the method consists of a sulfuric acid digestion step followed by dilution and hydrolysis by autoclaving, then HPLC analysis of the dissolved sugars. Gravimetric analysis of the solid residue followed by combustion was used to determine the acid-insoluble (Klason) lignin and ash contents. For analysis of pretreated pulps, sugar analysis was not completed and only the acid-insoluble lignin and ash contents were determined. Klason analysis was conducted in triplicate on pretreated air-dried pulps. Results of all biomass compositional and Klason analyses are reported on a dry weight basis. The untreated biomass lignin and pulp lignin content, both determined by compositional analysis, were used to calculate the pulp delignification. Further details are mentioned in Additional file 1.

### Particle size distribution measurements

Particle size distributions of untreated air-dried long fiber bagasse, short fiber bagasse and pith bagasse were determined by shaking on a vibratory sieve shaker (Retsch AS 200, Germany) equipped with stacked sieves (pore sizes between 850 and 180 μm) for 20 min. The percentage weight of material retained by each sieve was measured, and from these data the log-normal distribution mass median diameter (*D*_50_) was calculated. Particle size analyses were carried out in monoplicate. More details regarding the method and the calculations can be found in Additional file 1.

### Wavelength-dispersive X-ray fluorescence (WD-XRF)

Ash (i.e., inorganic material) samples (~ 1 g) were obtained from whole bagasse, depithed bagasse and pith bagasse by heating to 575 °C to remove any organic material in a programmable muffle furnace. The composition of the ash obtained was analyzed by quantitative WD-XRF, which was carried out by ITRI Innovation Ltd (UK). The full method is presented in Additional file 1.

### Scanning electron microscopy (SEM)

The morphologies of untreated and pretreated long fiber bagasse and pith bagasse samples were visually observed using scanning electron microscopy (JEOL JSM-5610 LV, JEOL Ltd, Japan). The pulp samples were mounted on a 1 cm^2^ metal sample holder using a carbon black sticker. The sample was then gold coated in an argon atmosphere using a sputter coating inside an EmiTech K550 (Emitech Ltd., Chelmsford, UK). Representative images of pretreated sugarcane bagasse were acquired with a 20 kV accelerating voltage at magnifications ranging from 50× to 500×.

## Results and discussion

### Compositions of the bagasse fractions

**Fig. 2:**
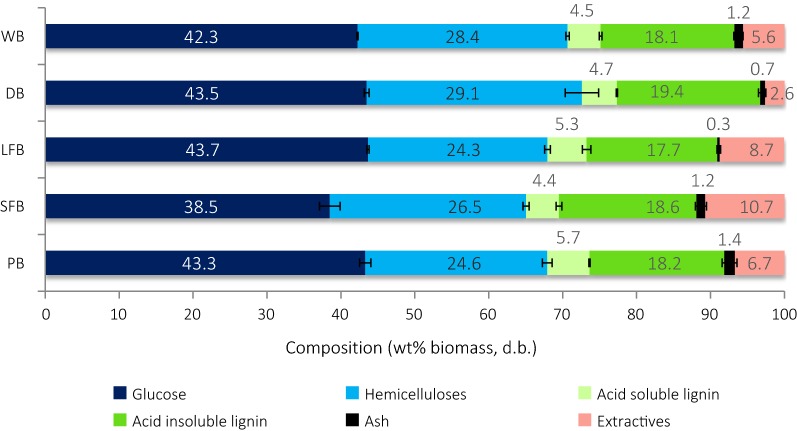
Composition of untreated bagasse fractions reported as a percentage of unextracted biomass. Error bars shown were calculated as the standard deviation across triplicates

Though depithing of bagasse is considered essential prior to paper and pulp making, its impact on biorefining has not yet been investigated. The performance of the novel ionoSolv pretreatment technology on five preparations of whole and depithed bagasse was investigated. Whole (WB), industrially depithed (DB), laboratory depithed long fiber (LFB) and short fiber bagasse (SFB) and pith bagasse (PB) preparations were subjected to compositional analysis prior to pretreatment. The compositions of the untreated materials are shown in Fig. 2 and their descriptions and appearances are found in Table 1.

**Table 1:** Lignocellulosic materials prepared from South African sugarcane bagasse used in this study and their appearance after milling and before pretreatment

Fraction	Description	Appearance
Whole bagasse (WB)	Heterogeneous mixture of longer fibers from within the sugarcane stalk cortex (fiber), short parenchymatous particles from the vascular bundle (pith) as well as the dense non-fibrous epidermis surrounding the stalk (rind)	
Depithed bagasse (DB)	Higher density, fibrous material left over after removal of pith particles by commercial depithing in an industrial process involving hammer milling of moist bagasse followed by screening	
Long fiber bagasse (LFB)	Longer fibers from the cortex of sugarcane stalk with high aspect ratios; retained on the first (12-mesh) screen within a wet depithing process involving agitation using water and air followed by sieving within an automated pith–fiber separator in the laboratory	
Short fiber bagasse (SFB)	Short fibers from the cortex of sugarcane stalk with moderate aspect ratios; separated by passing through the first (12-mesh) and being retained by the second (20-mesh) screen within a wet depithing process involving agitation using water and air followed by sieving within an automated pith–fiber separator in the laboratory	
Pith bagasse (PB)	Small, spongy spherical particles from the vascular bundle of sugarcane stalk mixed with short bagasse fibers. Lower density, porous material; obtained by passing through the second (20-mesh) screen within a wet depithing process involving agitation using water and air followed by sieving within an automated pith–fiber separator in the laboratory	

Chemical analysis revealed that the lignin content of South African sugarcane bagasse fractions (23–26% on an extractives-free basis) is higher than that of sugarcane bagasse feedstocks originating from other geographies. Other authors [13, 62] reported lignin contents of 17% and 18% for Mexican and Chinese bagasse, respectively. However, comparing the five bagasse South African preparations did not show substantial differences in their chemical compositions, as expected from the literature [13]: similar contents of total lignin (23–24%), carbohydrates (65–72%) and ash (0.3–1.4%) were seen relative to the biomass dry weight. More strikingly, there was no significant difference between the ash content of the pith (1.4 ± 0.0%) and whole bagasse (1.2 ± 0.3%). This finding is in disagreement with literature, which reports that pith particles contain more ash than fibrous bagasse [63] and where total ash contents in whole bagasse of 1–5% have been reported [13]. This suggests that pith and inorganic material adhering to the pulp were removed by washing during biomass preparation. Ash-rich pith particles may also have been lost in the sub-0.18 mm fraction during particle size standardization, reducing the ash content detected. Finally, we note that laboratory depithed fractions do not originate from the same whole biomass sample as industrially depithed bagasse, as observed in their higher extractives content.

### Optimal conditions for whole and industrially depithed bagasse

Whole and industrially depithed bagasse preparations were first pretreated with a [TEA][HSO_4_]:H_2_O (4:1 w/w) solution at 10 wt% biomass loading and fractionated into (1) a cellulose-rich material (pulp), (2) a lignin precipitate and (3) the recovered IL solution.

**Fig. 3:**
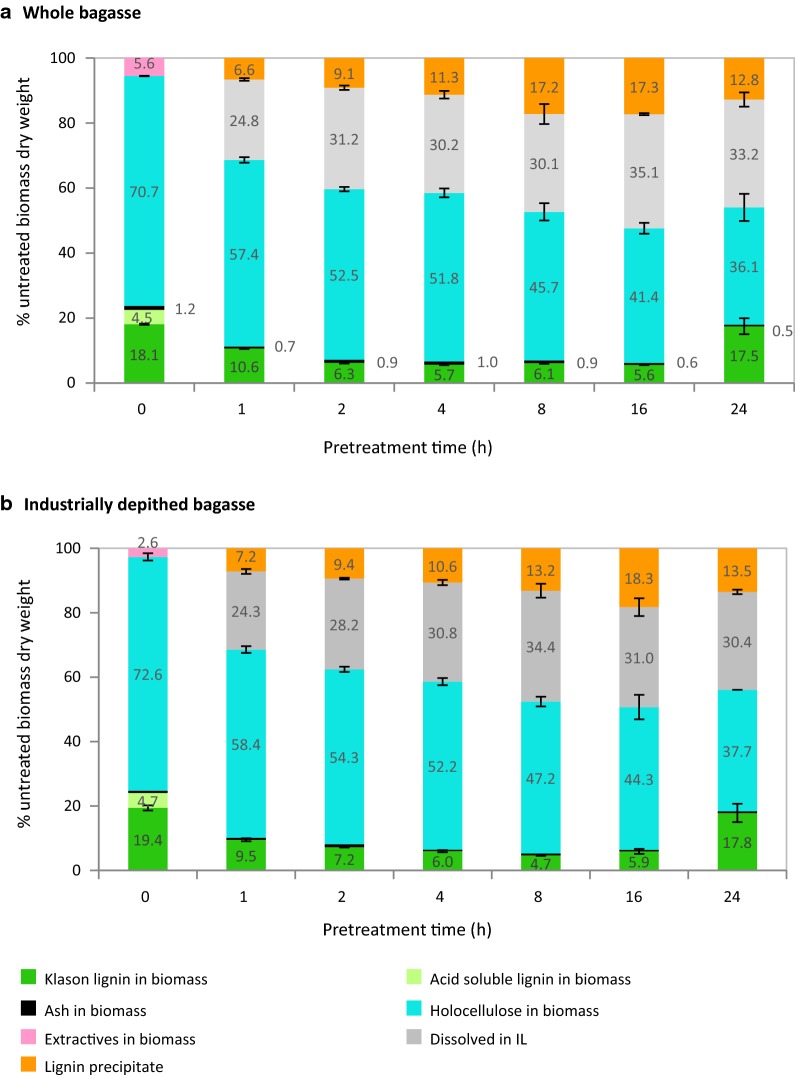
Untreated bagasse and recovery of pulp components, lignin precipitate and components dissolved in IL as a function of pretreatment time at 120 °C shown for **a** whole bagasse and **b** industrially depithed sugarcane bagasse. Pretreatment employed [TEA][HSO_4_]:[H_2_O] (4:1 w/w) with solids loading of 10% (w/v)

The optimum pretreatment condition was determined by varying the residence time between 1 and 24 h at 120 °C. Figure 3 shows the yields of components recovered in the pulp, lignin precipitate and dissolved in the IL, as a proportion of untreated material. Pulp yields decreased smoothly with longer treatment time, indicating progressive dissolution of hemicelluloses and lignin into the IL, as reported previously for biomass feedstocks treated with the same PIL [32, 57]. In our previous study of the hemicellulosic fraction in *Miscanthus* [32], hemicelluloses were seen to be extracted into the ionic liquid, where they hydrolyzed into monomeric sugars and underwent further dehydration and other decomposition reactions, forming furfural, 5-hydroxymethylfurfural, and levulinic and acetic acids. *Miscanthus* is chiefly composed of xylose with minor components of arabinose and galactose [32], while sugarcane bagasse hemicelluloses comprise mostly xylose with small amounts of arabinose and mannose. Though the IL solutions were not analyzed in this study, given that the PIL and pretreatment temperature used were the same, the behavior of the hemicellulosic fraction in both feedstocks is expected to be very similar.

Bagasse lignins precipitated from IL solutions have been analyzed by HSQC NMR and have been shown to be of a high purity, as evidenced by the absence of carbohydrate signals after 4 h of treatment (Figure S1 in Additional file 1); this is in accordance with results obtained in our previous studies of lignins isolated from similar grassy feedstock *Miscanthus* [55]. Lignin recovery increased with treatment time; the exception was at 24 h, where the lignin yield decreased while the Klason lignin detected in the pulp increased. This increase of lignin detected in the pulp is in agreement with studies of *Miscanthus* under severe pretreatment conditions, such as long residence time, high temperature and high acidity [32, 41, 53]. It is evidence for the formation of highly condensed lignins or insoluble carbohydrate degradation products called “pseudo-lignin”. These undesirable products are indistinguishable from each other using the Klason lignin detection method used to analyze the pulp composition [32] and are difficult to analyze by conventional analytical techniques due to their insolubility. However, strong evidence for the formation and partial re-deposition onto the pulp of condensed lignins and pseudo-lignins can be found in our prior works [32, 53, 55].

**Fig. 4:**
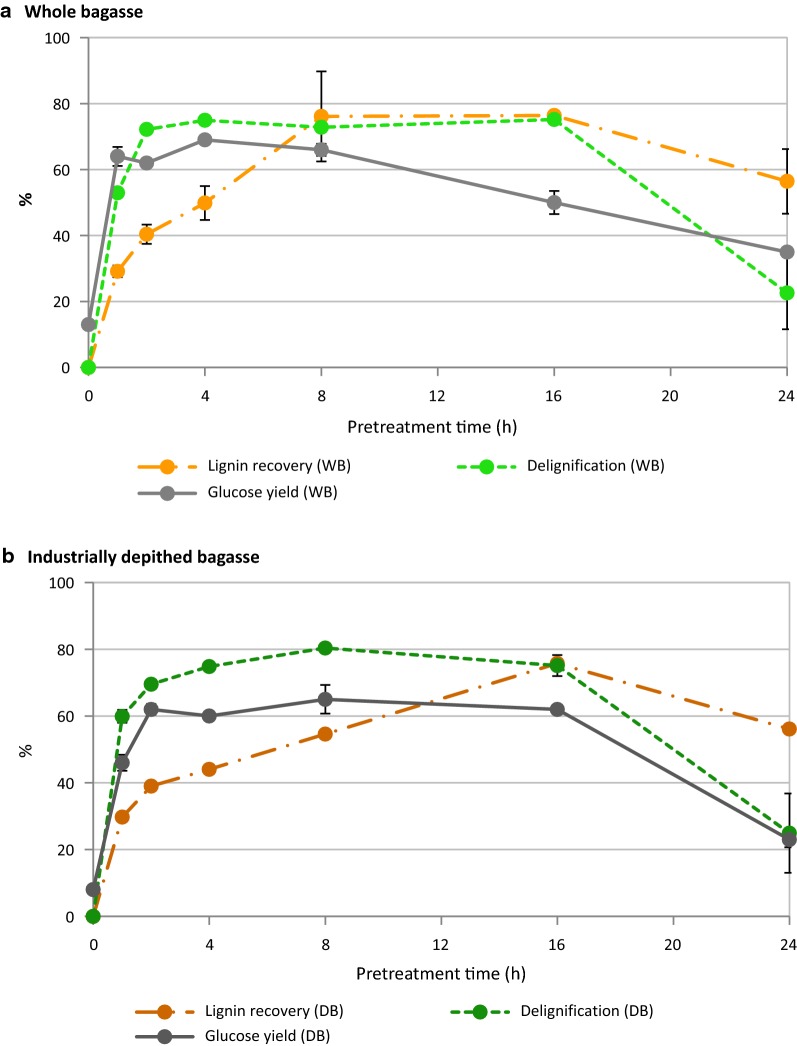
Key pretreatment indicators for **a** whole bagasse and **b** industrially depithed sugarcane bagasse pretreatment at 120 °C using [TEA][HSO_4_]:[H_2_O] (4:1 w/w) with solids loading of 10% (w/v)

Key indicators of pretreatment effectiveness, namely the lignin recovery, pulp delignification and glucose yield from enzymatic hydrolysis, are plotted in Fig. 4. For whole bagasse, delignification (i.e., removal of lignin) proceeded rapidly, reaching 75% within only 4 h of treatment. Maximum lignin removal coincided with maximum glucose yield (69 ± 1%). Industrially depithed bagasse showed very similar trends, though the pulp was most delignified (81%) after 8 h of treatment and glucose release from this pulp reached 65 ± 1%. These findings indicate rapid fractionation of sugarcane bagasse during pretreatment with [TEA][HSO_4_], with peak glucose release and high lignin removal from the pulp after 4 h of treatment at 120 °C. Beyond this point, the time course clearly shows that when lignin recovery appears to exceed delignification after 16 h, the glucose yield dropped. This observation strongly suggests the formation and deposition of condensed lignin or pseudo-lignin onto the pulp surface, impeding access by hydrolytic enzymes, as previously observed during ionoSolv processing of *Miscanthus*, willow and pine [32, 41, 53, 54, 64].

**Fig. 5:**
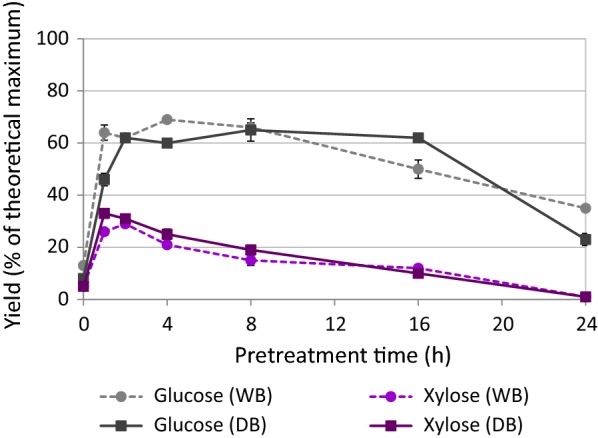
Comparison of glucose and xylose yields after 7-day enzymatic saccharification for whole bagasse (WB) and industrially depithed (DB) sugarcane bagasse pulps following pretreatment in [TEA][HSO_4_]:[H_2_O] (4:1 w/w) with solids loading of 10% (w/v)

Comparison of the enzymatic glucose and xylose release from whole and depithed bagasse (Fig. 5) revealed that commercial depithing did not have any significant effect on improving sugar yields. These results agree with the findings of Hernández-Salas et al. [26], who observed similar sugar yields from depithed and pith bagasse following acid hydrolysis, enzymatic hydrolysis, and dilute alkaline pretreatment plus enzymatic hydrolysis. This is in alignment with the similar compositions of the bagasse preparations (Fig. 2) and the fact that particle sizes were standardized within a 0.18–0.85 mm size fraction before pretreatment (given that particle size affects pretreatment outcomes). Xylose yields peaked first, reaching ~ 30% following only 1–2 h of pretreatment for both materials. Strikingly, glucose yields also attained high levels (more than 90% of the maximum glucose release) within 1–2 h of treatment; in a commercial scenario, the treatment would likely be limited to this point to save on reactor and operating costs. Maximal glucose yields of 69 ± 1% and 65 ± 4% glucose release were obtained under optimal conditions (120 °C, 4 and 8 h, respectively) for whole and depithed bagasse, respectively. These peak glucose yields obtained using low-cost [TEA][HSO_4_]–water mixtures were ~ 75% as high as those obtained by George et al. [38] using the APIL [Emim][OAc] for pretreatment of switchgrass, a grass feedstock with similar composition to bagasse. This result is highly promising given that [TEA][HSO_4_] has 1/40th the production cost and is much more thermally stable than [Emim][OAc] [38, 51]. Our findings closely match with Rocha et al. [44] result of 68% maximum glucose yield using the PIL [H_3_N(CH_2_)_2_OH][OAc]:[H_2_O] (5:1 w/w) at the same biomass loading, but the authors could not maintain this conversion efficiency for more than one PIL use cycle. In contrast, the [TEA][HSO_4_]:H_2_O (4:1 w/w) solution used in this study has been successfully recycled four times without major loss in conversion efficiency, as demonstrated by Brandt et al. [32]. We note that the incomplete digestion of the pretreated pulp is not likely to be a waste disposal problem in the sugarcane industry, as the post-hydrolysis solids could be dried and burned in a boiler to help meet the energy requirements of the mill or ethanol plant.

### Comparison of industrial and laboratory depithed preparations

This study also looked at bagasse preparations obtained from a laboratory-scale automated fiber–pith separator, in order to examine pretreatment performance on pith itself. Sieving bagasse in the laboratory has been reported to remove up to 25% more pith than commercial depithing [22], presumably because it uses two screening stages and gentler fiber separation using water and air rather than rotating hammers. The laboratory depithing method produced three bagasse fractions, namely long fiber, short fiber and pith bagasse, which were ground and sieved to 0.18–0.85 mm before pretreatment.

**Fig. 6:**
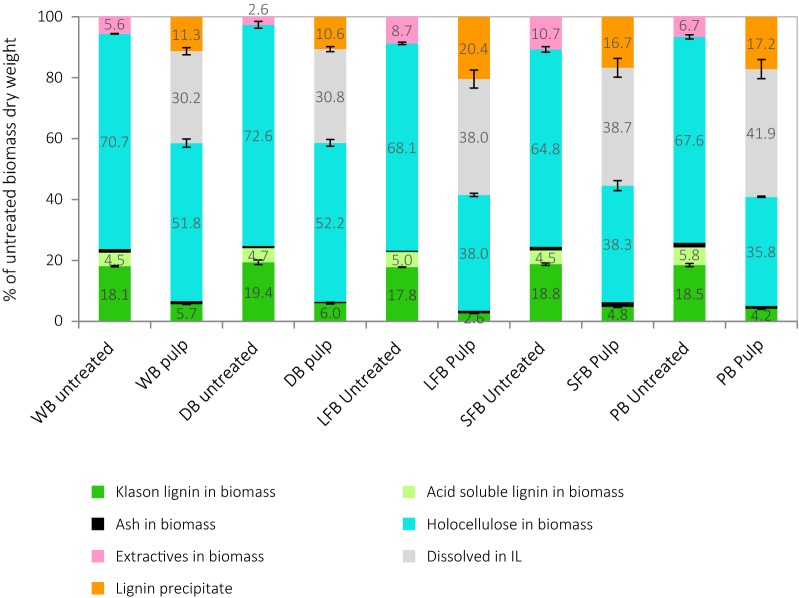
Mass balance of untreated bagasse and recovered pulp, lignin precipitate and components dissolved in IL after pretreatment at 120 °C for 4 h, shown for whole, industrially depithed and laboratory depithed sugarcane bagasse fractions

Pretreatment of three bagasse preparations obtained from laboratory separation was compared with whole and industrially depithed bagasse at the optimum condition of 120 °C and 4 h, under otherwise identical treatment conditions. The mass balance of pulp, lignin and dissolved components is shown in Fig. 6. The five fractions underwent fractionation in a similar fashion. Greater removal of lignin and hemicellulose into the IL was seen for laboratory depithed materials and pith bagasse, which also produced more lignin precipitate, suggesting lignin extraction was more rapid and the solubility of the extracted lignin in water was lower, causing more of it to precipitate upon addition of anti-solvent.

**Fig. 7:**
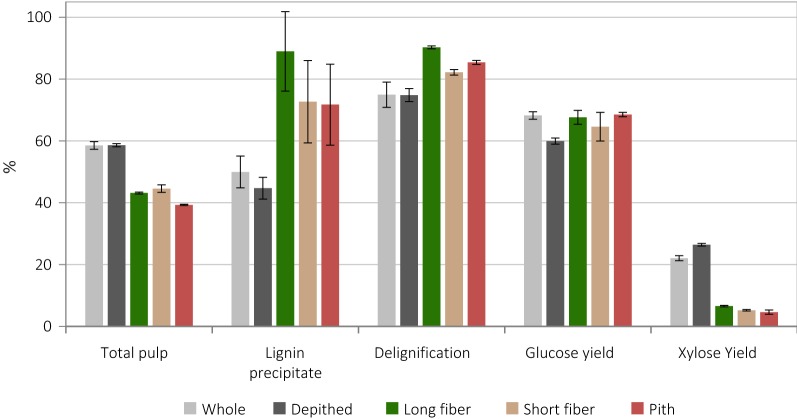
Pretreatment outcome for whole, depithed and laboratory depithed fractions of sugarcane bagasse following pretreatment at 120 °C for 4 h, reported as in previous figures. NB. Delignification reported as a % of acid-insoluble lignin remaining in pulp compared to total (acid-soluble + acid-soluble) lignin in untreated biomass

The delignification from the pulp was calculated from Klason analysis, and is presented together with the pulp and lignin recovery, glucose and xylose yield for all five preparations (Fig. 7). Under the same pretreatment conditions, significantly more lignin was isolated from fractions obtained by laboratory depithing (72–89%) compared to whole and industrially depithed bagasse (45–50%). High lignin recovery was also accompanied by lower pulp yields and slightly enhanced delignification of the pulp, which approached 90% for laboratory depithed fibers and the pith. This indicates that both a larger proportion of lignin was extracted into the IL and also recovered as a precipitate. Given the similar compositions of all untreated biomass preparations (Fig. 2), these findings could be explained either by differences in particle morphology or in native lignin structure (between WB/DB and LFB/SFB/PB, which were sourced separately); both are discussed further below.

It is noteworthy that long fiber bagasse was the most delignified and gave the highest lignin yield among the laboratory depithed fractions. This may be explained by the lower surface area to volume ratio of long fibers compared with shorter fibers, reducing lignin re-deposition onto the pulp surface (see also Table 1). To assess the possibility of particle size effects, the particle size distributions of the untreated fractions were measured (see Figure S2 in Additional file 1) and the geometric average particle size *D*_50_ was calculated. Long fiber and pith bagasse had very similar particle size distributions and a geometric average particle size *D*_50_ of around 0.4 mm, while short fiber bagasse appeared to have slightly larger particles on average, with *D*_50_ of 0.55 mm. However, the (similar) particle size distributions did not reveal any differences in aspect ratio, which could not be measured in this study due to limited sample size [65]. Careful consideration of the effect of particle size distribution, aspect ratio and specific surface area is, therefore, recommended for future studies.

The results of enzymatic hydrolysis reveal that glucose yields from pretreated bagasse pulps were very similar for the five preparations, ranging between 60 and 69% of the theoretical maximum release. No improvement in glucose yield was seen for long fiber bagasse though it produced the most delignified pulp. Indeed, the glucose yields reflect the similar glucan and lignin contents of the starting materials (Fig. 2). Neither industrial nor laboratory depithing had a significant effect on improving glucose release from pretreated bagasse pulps. However, xylose yields for laboratory depithed materials were significantly lower (< 5%) compared to those for whole and industrially depithed bagasse (22% and 26%, respectively). It is likely that hemicellulose release was limited by hemicellulose content remaining in the pulp, from where it may have been removed more rapidly during pretreatment of laboratory depithed material and pith bagasse, which also saw more rapid delignification. This is supported by the lower pulp yields observed for these materials (Fig. 6).

As noted previously, laboratory depithed materials originated from a different bagasse harvest as whole and industrially depithed bagasse. Despite the similar chemical compositions of the untreated bagasse preparations (Fig. 2), there may be differences in native lignin structure influencing biomass recalcitrance. This may contribute to explaining the higher yield of lignin precipitate obtained for fractions separated in the laboratory, especially long fiber bagasse (89%). Size exclusion chromatography (SEC) of the lignins extracted during pretreatment of industrially depithed and long fiber bagasse was performed to compare their molecular size. The results (Figure S3; see Additional file 1) revealed a larger proportion of high molecular mass material in long fiber bagasse lignin compared to depithed bagasse lignin. This indicates that lignin isolated from long fiber bagasse is likely to have a higher average molecular weight than depithed bagasse lignin. The lower solubility of high molecular weight lignin fragments extracted from long fiber bagasse is proposed as a major reason for its high lignin precipitate recovery. However, this may also delay lignin extraction into the IL solution by ether bond cleavage, a possible explanation for the smaller difference between delignification (lignin removal) and lignin precipitation seen in laboratory separated fractions compared to whole and industrially depithed bagasse, with lower delignification limiting glucose yields.

To further investigate the influence of fiber morphology on pretreatment outcome, the microstructural features of untreated and pretreated long fiber and pith bagasse were visually examined using scanning electron microscopy (SEM) (see Figure S4 in Additional file 1). Long fiber bagasse is made up of long cellulose fibers, while pith bagasse has a rough porous structure made up of tubular openings known as “lumen” that gives it very high specific surface area [65, 66].

Based on SEM images, the surface morphologies of pith and long fibre bagasse after 4 h of PIL treatment did not show any significant differences compared to their respective parent biomass. This is in contrast to the severe disruption of biomass surfaces observed following treatment with cellulose-dissolving APILs such as [Emim][OAc] [67], which have the property of disrupting the crystalline structure of cellulose. We note that lignin deposition onto the pulp surface, which is strongly suspected to occur under prolonged treatment as discussed above, is unlikely to be observed in these SEM images due to the short treatment time used. However, strong evidence of this surface layer that would retard enzymatic hydrolysis was found in recent work by Gschwend et al. [53]. Therefore, the specific surface area of particles is believed to be a major factor in bagasse hydrolysis owing to the greater likelihood of lignin precipitation onto particles with higher surface area, such as pith bagasse.

### Mass balance

**Fig. 8:**
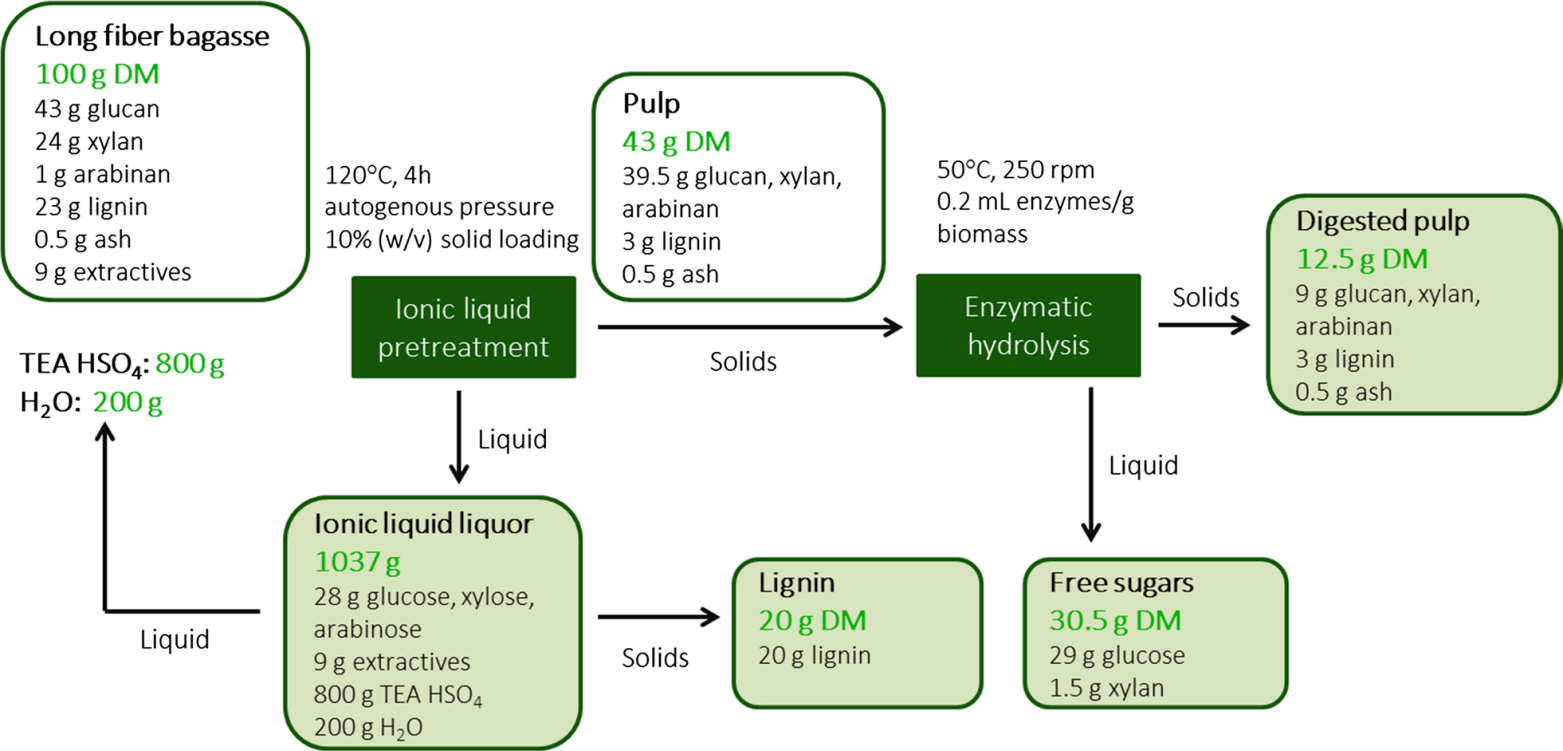
Mass balance during ionoSolv pretreatment of long fiber sugarcane bagasse in [TEA][HSO_4_]:[H_2_O] (4:1 w/w) at 10% (w/w) solids loading at 120 °C for 4 h and subsequent enzymatic hydrolysis at 1% (w/v) solids using NS22201 enzymes. The calculations assume 100% ionic liquid recovery. *DM* dry mass

Mass balances are essential when performing techno-economic and life cycle analyses of biorefining processes to ensure holistic biomass utilization and minimal waste generation. An overall mass balance of the ionoSolv process applied to long fiber bagasse is presented in Fig. 8. For every dry mass unit of bagasse input into the process, we obtain 43 wt% as pulp, 20 wt% as lignin and the remaining 37% is found dissolved in the IL solution. We note that ionoSolv processing was able to extract high lignin yields of ~ 3× the amount obtained by Rocha et al. [44] (6.5 g lignin/100 g bagasse) even while optimizing glucose release, which is advantageous for deriving additional value from bagasse. The pulp was subjected to enzymatic hydrolysis, whereby ~ 70% of the glucan and xylan polymers present in untreated bagasse were released as glucose and xylose (equivalent to ~ 30 g/100 g untreated biomass saccharified). 29.0 g glucose was obtained from 100 g of untreated bagasse, which is slightly less than Rocha et al.’s [44] finding of 35.5 g glucose/100 g bagasse. The post-hydrolysis solids left behind contained roughly 25 wt% lignin. It is anticipated that the post-hydrolysis solids would be dried and combusted to contribute to the energy requirements of the ethanol plant or sugarcane mill, the implications of which are discussed further below.

### Ash analysis

Presently, about 50 wt% of the bagasse produced in the sugarcane industry is burned, producing significant quantities of fly ash [11]. Understanding the composition of the inorganic components in bagasse is thus relevant for their sustainable disposal and/or valorization. As in a previous study of the ionoSolv process using *Miscanthus* [32], it was found that the majority of ash partitioned with the pulp (see Additional file 2). The post-hydrolysis solids would thus be expected to contain most of the inorganic material in bagasse; this solid residue is also a likely fuel source in the sugarcane or ethanol mill. The chemical composition of bagasse ash is also of particular interest for combustion and pyrolysis applications [19, 68, 69].

Industrially depithed bagasse, long fiber bagasse and pith bagasse were heated in a muffle furnace at 575 °C to obtain ash which is free of untrapped or unburnt carbon. The ash samples were analyzed by wavelength-dispersive X-ray fluorescence (WD-XRF) (Table 2). Silica was found to be the major component, accounting for roughly half of depithed and long fiber bagasse ash and over 70% of pith ash by weight. Other major components were aluminum, calcium, iron and magnesium oxides, agreeing with the literature reports [70]. The high silica component of bagasse is well known in the industry [18] and is among the reasons for pith removal from bagasse prior to paper pulping [63]; however, there is no evidence that depithing improves combustion performance other than that depithed bagasse retains less water [22].

**Table 2:** Composition of depithed bagasse ash, long fiber bagasse ash and pith ash as determined by WD-XRF

Compound (wt%)	Depithed bagasse (DB) ash	Long fiber bagasse (LFB) ash	Pith bagasse (PB) ash
SiO_2_	50.8	54.6	70.1
Al_2_O_3_	3.6	0.5	1.1
Fe_2_O_3_	6.6	0.7	1.4
Mn_3_O_4_	0.2	0.3	0.1
SO_3_	1.3	2.2	1.8
CaO	8.9	11.2	13.1
MgO	4.5	10.2	5.2
ZnO	0.07	0.08	0.06
Na_2_O	7.3	4.4	5.0
K_2_O	1.6	9.6	1.00
P_2_O_5_	2.9	6.0	1.7
TiO_2_	0.3	0.09	0.2
SrO	0.05	0.05	0.05
BaO	< 0.01	0.08	0.09
LOI	12	N/A	N/A

*LOI* loss on ignition

## Conclusions

[TEA][HSO_4_]:H_2_O (4:1 w/w) solutions were shown to efficiently fractionate whole, depithed, fibrous and pith bagasse into a cellulose-rich pulp and a lignin precipitate. High purity cellulose pulps with up to 90% lignin removal and 69% glucose release were obtained, i.e. ~ 75% of the glucose released using expensive [Emim][OAc] as solvent, a promising result given [TEA][HSO_4_] is 40 times less expensive. The results suggest that low-cost acidic ionic liquid solutions are promising candidate solvents for large-scale processing of raw sugarcane bagasse.

Using a room temperature laboratory separation of pith and fibrous fractions in bagasse appears to be useful where lignin extraction is a key objective. However, where a high fermentable sugar recovery is paramount, as in most biofuels production processes, depithing seems to be an unnecessary step. Avoiding the high energy input and equipment costs of the depithing operation, estimated to be around US$5.50/ton of bagasse [16], may also contribute to lowering capital and operating costs, helping to accelerate the development of cost-effective biorefining processes using sugarcane bagasse. Depithing also has implications for the bulk density, water-holding capacity and flammability of stockpiled bagasse, which need to be investigated prior to recommending the use of whole non-depithed bagasse as a feedstock for a commercial-scale biorefinery.

## Additional files

**Additional file 1: Figure S1.** HSQC NMR spectra (side chain region) of lignins isolated from depithed sugarcane bagasse (DB) using [TEA][HSO_4_] containing 20 wt% water and a solids loading of 20 wt% at 120 °C for 4 and 8 h. Representative substructures are shown. **Figure S2.** Particle size distribution of untreated pith (PB), long fiber (LFB) and short fiber bagasse (SFB) obtained after sieving within the pretreatment size fraction (0.18–0.85 mm). Inset values show calculated geometric mean particle length D50. **Figure S3.** Area-normalized size exclusion chromatograph of ionoSolv lignins isolated from industrially depithed (DB) and long fiber bagasse (LFB) pretreated in [TEA][HSO_4_] containing 20 wt% water at 120 °C for 4 h with 10 wt% solids loading. Mixed-D column, NMP eluent, 300 nm. **Figure S4.** Scanning electron micrographs of longitudinal view of, **a** pith bagasse before pretreatment, **b** pith bagasse after pretreatment, **c** long fiber bagasse before pretreatment and, **d** long fiber bagasse after pretreatment. Treatments were conducted in [TEA][HSO_4_] containing 20 wt% water and solids loading of 10 wt% at 120 °C for 4 h

**Additional file 2: Table S1.** Chemical composition analysis of untreated South African sugarcane bagasse preparations used in this study. **Table S2.** Pretreatment outputs.
