# Supplementary material for: Pretreatment of South African sugarcane bagasse using a low-cost protic ionic liquid: a comparison of whole, depithed, fibrous and pith bagasse fractions
Source: Biotechnol Biofuels. 2018 Sep 11;11:247. doi: 10.1186/s13068-018-1247-0 (PMC6131805; doi:10.1186/s13068-018-1247-0)
Supplement: Supplementary file 1 — Additional file 1: Figure S1. HSQC NMR spectra (side chain region) of lignins isolated from depithed sugarcane bagasse (DB) using [TEA][HSO4] containing 20 wt% water and a solids loading of 20 wt% at 120 °C for 4 and 8 h. Representative substructures are shown. Figure S2. Particle size distribution of untreated pith (PB), long fiber (LFB) and short fiber bagasse (SFB) obtained after sieving within the pretreatment size fraction (0.18–0.85 mm). Inset values show calculated geometric mean particle length D50. Figure S3. Area-normalized size exclusion chromatograph of ionoSolv lignins isolated from industrially depithed (DB) and long fiber bagasse (LFB) pretreated in [TEA][HSO4] containing 20 wt% water at 120 °C for 4 h with 10 wt% solids loading. Mixed-D column, NMP eluent, 300 nm. Figure S4. Scanning electron micrographs of longitudinal view of, a pith bagasse before pretreatment, b pith bagasse after pretreatment, c long fiber bagasse before pretreatment and, d long fiber bagasse after pretreatment. Treatments were conducted in [TEA][HSO4] containing 20 wt% water and solids loading of 10 wt% at 120 °C for 4 h [file 13068_2018_1247_MOESM1_ESM.docx]

**Additional file 1**

**Ionosolv fractionation protocol**

The solvent i.e. ionic liquid-water solution used in the pretreatment process was a mixture of 80 wt% [TEA][HSO_4_] and 20 wt% water. 1.00±0.05 g air-dry biomass was placed in a wide-mouthed 15 mL pressure-resistant glass tube with screw-on Teflon cap and O-ring seal (Ace Glass #8648-04 tube with #15 front-seal plug, Vineland, NJ, USA). 10.00±0.05 g of ionic liquid solution was added, the tubes sealed and the contents mixed with a vortex shaker. The samples were then placed into a preheated convection oven (HeraTherm OMH60, ThermoScientific) pre-heated to 120 °C for between 1 and 24 hours. After the pretreatment period, the tubes were removed from the oven and allowed to cool to room temperature. Experiments were carried out in triplicate.

After the pretreatment, the pulp/ionic liquid mixture were washed four times with ethanol (40 mL each). The ionic liquid/ethanol mixture and pulp were separated by centrifugation at 4,000 rpm for 50 min. The solids were re-suspended in fresh ethanol after each washing step and the supernatant was consecutively collected. The carbohydrate-rich material (solids or ‘pulp’) was transferred into a cellulose thimble and Soxhlet extracted with refluxing ethanol (150 mL, 24 h). The thimbles were then left to air-dry on the bench overnight. The Soxhlet extraction washings were combined with the ethanol washings from the previous steps and evaporated *in vacuo* at 40 °C, leaving a dried ionic liquid/lignin mixture. Lignin was precipitated by adding 40 mL of deionized water to this mixture. The precipitated lignin was isolated by centrifugation as above and the supernatant decanted and collected. Lignin washing was repeated three more times. The lignin precipitate was dried *in vacuo* at 45 °C for 24 h. The air-dried pulp yield was determined by weighing the recovered biomass from the cellulose thimble and the oven-dried weight was determined. The dried lignin yield was recorded.

The air-dried pulp yield was calculated according to the following equation:

$Y_{pulp}\left( \% \right)=\frac{{ODW}_{pulp}}{{ODW}_{feedstock}}\times100=\frac{m_{pulp}\left( 100-{mc}_{pulp} \right)}{m_{feedstock}\left( 100-{mc}_{feedstock} \right)}\times100$ (Equation S1)

where ODW is the oven-dried weight of material, *m*_pulp_ is the mass of air-dried pulp recovered after the Soxhlet step, *mc_pulp_* is the pulp moisture content (wt%), *m_feedstock_* is the amount of air-dried untreated biomass added to the pretreatment tube, and *mc_feedstock_* is the moisture content of the feedstock (wt%).

The lignin yield was calculated according to the following equation:

$Y_{lignin}\left( \% \right)=\frac{{ODW}_{lignin}}{{ODW}_{feedstock}}\times100=\frac{m_{lignin}}{m_{feedstock}\frac{\left( 100-{mc}_{feedstock} \right)}{100}}\times100$ (Equation S2)

where, *m*_lignin_ is the oven-dried mass of lignin precipitate recovered.

**Enzymatic hydrolysis assay**

Hydrolysis assays were carried out in triplicate with blanks (also in triplicate). 100±10 mg (calculated on an oven dried weight basis) of air-dried biomass or pulp was placed in a 30 mL Sterilin container and the weight recorded. Three blanks were run with 100 *μ*L of purified water in order to correct for sugar residues present in the enzyme solutions. 9.9 mL solution consisting of 5 mL 0.1 M sodium citrate buffer at pH 4.8, 40 *μ*L tetracycline T7660 solution (10 mg/ml in 70% ethanol), 30 *μ*L of cycloheximide C7698 (10 mg/mL in purified water), 4.71 *μ*L purified water and 20 *μ*L enzyme solution (NS22201, Novozymes) were added to each sample, the tubes sealed and placed in a rotary shaker-incubator (New Brunswick Scientific Innova®42, USA) at 50 °C and 250 rpm for 7 days and the NS22201 was determined to have a specific activity of 70 ± 12 *μ*mol min^-1^ ml^-1,^ determined using an enzyme concentration assay based on the Bradford assay (BioRad, USA). After the hydrolysis period had elapsed, 1 mL of solution was filtered through a 0.2 *μ*m PTFE syringe filter (VWR). Samples were run on a Shimadzu Prominence HPLC equipped with a refractive index detector, deashing cartridges and an Aminex HPX-87P column (Biorad, 300 x 7.8 mm, prepacked HPLC carbohydrate analysis column) with purified water as mobile phase (0.6 ml min^-1^). The column temperature was 85 °C and the acquisition time was 40 min. Calibration standards with concentrations of 0.1, 1, 2 and 4 mg mL^-1^ of glucose, xylose, mannose, arabinose and galactose and 8 mg mL^-1^ of glucose were used.

Glucose and xylose conversion was calculated based on the glucan and xylan content of untreated biomass determined by compositional analysis, and results are presented as a percentage of the theoretical maximum sugar release. To calculate glucose and xylose yields, the sugar concentrations obtained from HPLC analysis were first corrected by subtracting the average concentration of the respective sugar from the enzyme blanks. The resulting glucose and xylose concentrations (mg/mL) calculated for each mixture can then be converted to anhydro-glucose and anhydro-xylose concentrations, respectively, by subtracting out the proportional weight added to each molecule by the water of hydrolysis (i.e. multiplying by 0.90 for glucose and by 0.88 for xylose) which will convert the concentrations (mg/mL) of sugars found in the hydrolysis mixture to the ‘concentrations’ of material removed from the polysaccharides. This ‘concentration’ must then be multiplied by the volume of digestate solution for each sample (10 mL) and divided by the mass of glucan and xylan found in the untreated material:

$Glucose yield (\%)=\frac{\frac{mg sugar}{ml}\times volume\times Y_{pulp}}{\frac{g glucan}{g untreated biomass}\times g biomass added}*0.90*100$ Equation S3

$Xylose yield (\%)=\frac{\frac{mg sugar}{ml}\times volume\times Y_{pulp}}{\frac{g glucan}{g untreated biomass}\times g biomass added}*0.88*100$ Equation S4

where mg/mL is from HPLC analysis, the volume is 86.73 ml, *Y_pulp_* is the pulp yield determined according to Equation S1, g polysaccharide/g biomass is from the compositional analysis of untreated material, g biomass added is the oven-dry weight of biomass added to the assay tube.

**Compositional and Klason analysis**

Compositional analysis of untreated extractives-free biomass was carried out following the NREL protocol ‘Determination of Structural Carbohydrates and Lignin in Biomass’, NREL/TP-510-42618 [43]. Analysis was carried out in triplicate. To summarize, solids remaining after two-stage acid hydrolysis were held at 105 °C overnight. The mass of the ash-only fraction was then determined by heating the solids to 575 °C for 4 h in a programmable muffle furnace (Nabertherm Controller P330 LT 5/13, Nabertherm GmbH, Germany). The acid-soluble lignin (ASL) or Klason lignin content of the sample was determined by measuring the UV absorption of the acid hydrolysis supernatant at 240 nm wavelength and an absorptivity of 25 L g^-1^cm^-1^. Total lignin was calculated as the sum of acid soluble and acid insoluble lignin. HPLC analysis of glucose, xylose, mannose, arabinose and galactose was performed on a Shimadzu HPLC with an AMINEX HPX-87P Column (Biorad, 300 x 7.8 mm, pre-packed HPLC column) with refractive index detection. The content of carbohydrates, lignin and ash were expressed as a fraction of the sum of all components (normalized to 100%). For analysis of pretreated pulps, sugar analysis was not completed and only the Klason lignin was determined; this method is known as ‘Klason’ analysis.

The pulp composition was used to calculate the delignification (Equation S5); this were used together with the glucose and xylose yields from enzymatic hydrolysis as the key indicators of pretreatment performance.

$Delignification (\%)=\frac{{Lignin}_{untreated}-{(Lignin}_{pulp}\times Y_{pulp})}{{Lignin}_{untreated}}$ Equation S5

where *Lignin_untreated_* is the lignin content in untreated sugarcane bagasse, *Lignin_pulp_* is the lignin content in the pulp and *Y_pulp_* is the oven-dried yield of pulp.

**Particle size distribution measurements**

Particle size distributions of untreated air-dried long fiber bagasse, short fiber bagasse and pith bagasse were determined for untreated pulps using nested sieves. The sample particle size distribution was obtained using a vibratory shaker (Retsch SM 200, Germany) equipped with stacked sieves with decreasing pore sizes (850, 710, 500, 300, 212 and 180 μm) by shaking on a vibratory sieve shaker for 20 minutes. The percentage weight of material retained by each sieve was measured, and from this data the log-normal distribution mass median diameter (*D_50_*) was calculated, i.e. the particle size that 50% of the sample is smaller than by mass when a log-normal distribution is fitted to the measurements. It is used herein as a measure of the average particle size of a biomass sample and was calculated by linear interpolation according to Equation S6:

$D_{50}=10^\left( \left( \log x_{2}-\log x_{1} \right)\times\left( \frac{50-y_{1}}{y_{2}-y_{1}} \right)+\log x_{1} \right)$ Equation S6

where *x_1_* and *x_2_* are the pore sizes of the sieves (in mm) which allowed just under and just over 50% of the sample to pass through by weight, respectively, and *y_1_* and *y_2_* are the percentages of material passing through the sieves.

Particle size analyses were conducted in monoplicate and percentage errors were assigned according to our previous analysis for *Miscanthus* pulp particles (results not shown), which found an average of 1% error for untreated biomass samples. Particle size distributions were plotted as weight percentages of samples passing through different sieve pore sizes (on a log scale) and geometric mean particle sizes *D_50_* were compared.

**HSQC NMR spectroscopy**

For HSQC NMR experiments of precipitated lignins, ca. 20 mg of lignin was dissolved in 0.25 mL of DMSO-d_6_ and the solution transferred to a Shigemi tube. HSQC NMRs were recorded on a Bruker 600 MHz spectrometer (pulse sequence hsqcetgpsi2, spectral width of 10 ppm in F2 (^1^H) with 2048 data points and 160 ppm in F1 (^13^C) with 256 data points, 16 scans and 1 s interscan delay). Spectra were analysed using MestReNova (Version 8.0.0, Mestrelab Research 2012). All spectra were referenced to the DMSO peak at 2.500 ppm (^1^H) and 39.520 ppm (^13^C). The position of the integration areas were selected according to literature (Brandt *et al.*, 2015). Our analysis focuses on the side chain region (δ*C*/δ*H* 50-90/2.5-5.8 ppm) of the spectra, which contains polysaccharides (δ*C*/δ*H* 60-85/3.2-4.2) associated with residual carbohydrates

**Size exclusion chromatography (SEC)**

SEC was performed on lignins isolated from depithed bagasse and long fiber bagasse using 1-methyl-2-pyrrolidinone (NMP). The NMP eluent was obtained from Rathburn Chemicals Limited, UK (peptide synthesis-grade, UV absorbance cutoff 265 nm). SEC experiments were performed using lignins isolated from depithed bagasse and long fiber bagasse using a Mixed-D column (5 µm particle size, 300 mm x 7.5 mm I.D.; Polymer Laboratories, UK) packed with polystyrene/polydivinylbenzene beads. The Mixed-D column was operated with 0.5 mL min^-1^ NMP as eluent at 80 °C with a Knauer M100 isocratic HPLC pump. A Perkin-Elmer LC 290 variable wavelength UV-absorbance detector was used and routinely set to 270 and 300 nm. The system was calibrated using linear polystyrene standards with M_P_ values of 580, 970, 2,960, 19,880, 185,400, 523,000 and 5,000,000. Three linear PS standards across with M_P_ values of 970, 2960 and 19,880 were eluted to verify the calibration prior to each set of SEC experiments. The calibration curve used was *y* = 0.4038*x*+10.632, R*^2^* = 0.9746*,* where *y* = log[molecular weight (Da)] and *t* = elution time (min), valid between *t* = 11–19 min. SEC experiments were performed in triplicate. Polystyrene does not represent the geometry of the highly branched lignin macromolecule, so the molecular weights reported bear the risk of a systemic error; however, the relative trends in molecular weight distribution observed are valid. The ratio between the excluded (*t =* 0–15 min) and retained (*t* = 15–30) peak areas, i.e. A_E/R_, was calculated by numerical integration of the UV-absorbance signal versus time in Microsoft Excel.

**Wavelength-dispersive X-ray fluorescence**

Ash (inorganic material) samples of ~1g were obtained from whole bagasse, depithed bagasse and pith bagasse and analysed by WD-XRF. Ash samples were produced by heating to 575 °C to remove any organic material in a programmable muffle furnace (Nabertherm Controller P330 LT 5/13). The heating program was as follows: 25 → 105 °C with a heating rate of 20 °C min^-1^, plateau of 30 min at 105 °C, 105 → 350 °C with a heating rate of 3 °C min^-1^, plateau of 30 min at 350 °C, 350 → 575 °C with a heating rate of 3 °C min^-1^, plateau of 1 h at 575 °C, and annealing to room temperature. The ash obtained was a pink/orange powder and its composition was analyzed by quantitative WD-XRF. Sample preparation was by fused bead using an automated FluXana Vulcan system (Fluxana GmbH, Germany) using a standard 10:1 ratio of flux:material. This was carried out by ITRI Innovation Ltd (UK). The fused bead was analyzed using a PANalytical AxiosmAX minerals wavelength dispersive X-ray fluorescence spectrometer (PANalytical, Netherlands) equipped with a 60 kV generator and a 4 kW Rh SST-mAX X-ray tube. Analysis was carried out using Omnian software package for semi-quantitative XRF.


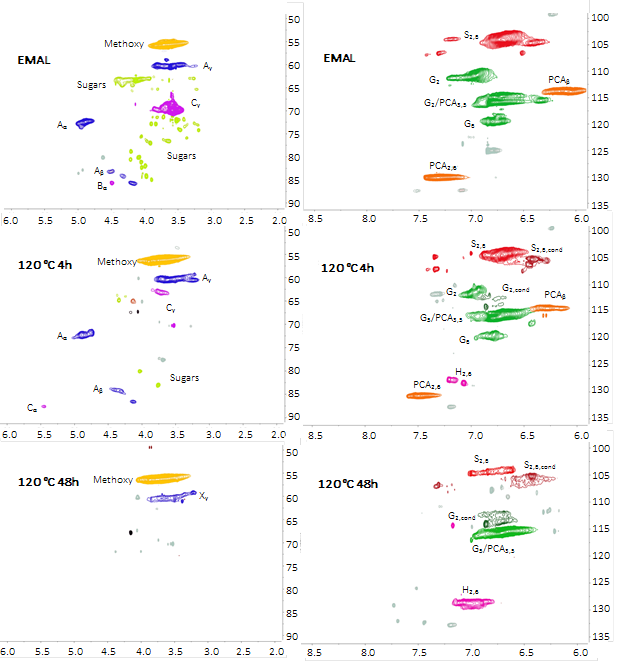


No sugars

Aγ

Methoxy

**120 °C 8 h**


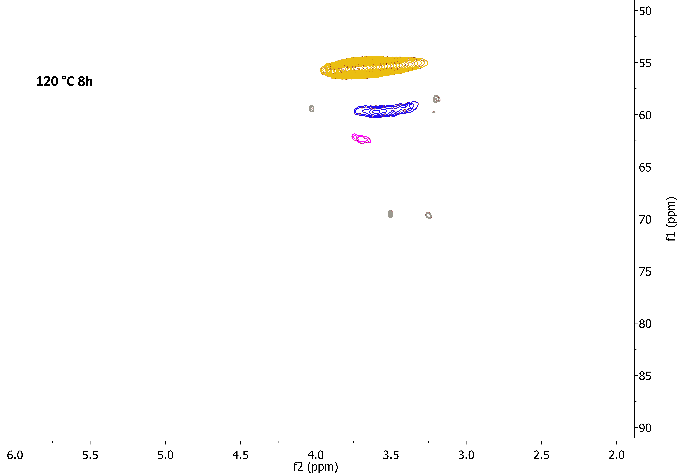

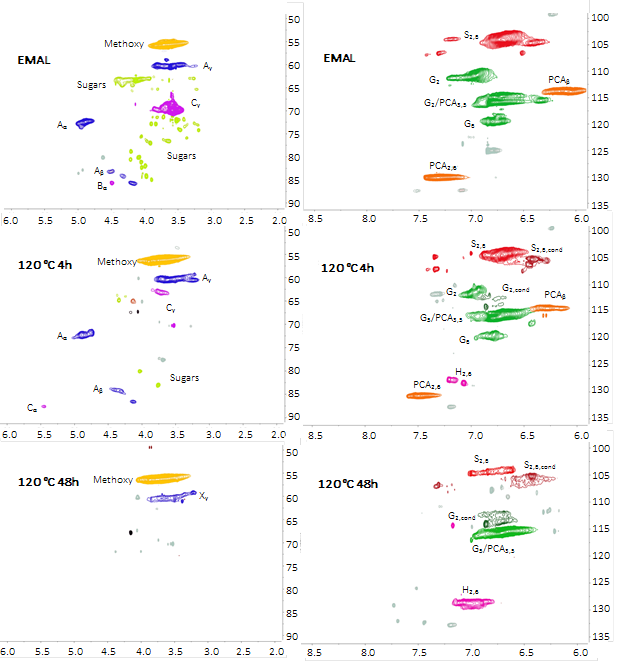

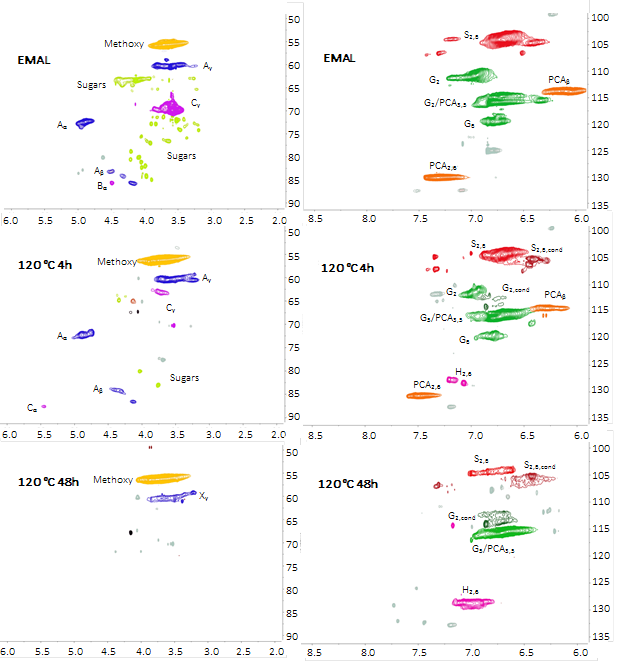





**Figure S1.** HSQC NMR spectra (side chain region) of lignins isolated from depithed sugarcane bagasse (DB) using [TEA][HSO_4_] containing 20 wt % water and a solids loading of 20 wt% at 120°C for 4 and 8 hours. Representative substructures are shown above.

The spectrum of lignin precipitated after 4 h of pretreatment can be seen to contain traces of polysaccharides (δ*C*/δ*H* 60-85/3.2-4.2) associated with residual carbohydrates. These correlations disappeared from the side chain region resulting in a carbohydrate-free lignin after 4 h of pretreatment.

**Figure S2.** Particle size distribution of untreated pith (PB), long fiber (LFB) and short fiber bagasse (SFB) obtained after sieving within the pretreatment size fraction (0.18-0.85 mm). Inset values show calculated geometric mean particle length *D_50_*.

**Figure S3.** Area-normalized size exclusion chromatograph of ionoSolv lignins isolated from industrially depithed (DB) and long fiber bagasse (LFB) pretreated in [TEA][HSO_4_] containing 20wt % water at 120°C for 4 h with 10 wt% solids loading. Mixed-D column, NMP eluent, 300 nm.

**c**

**d**

**b**

**a**


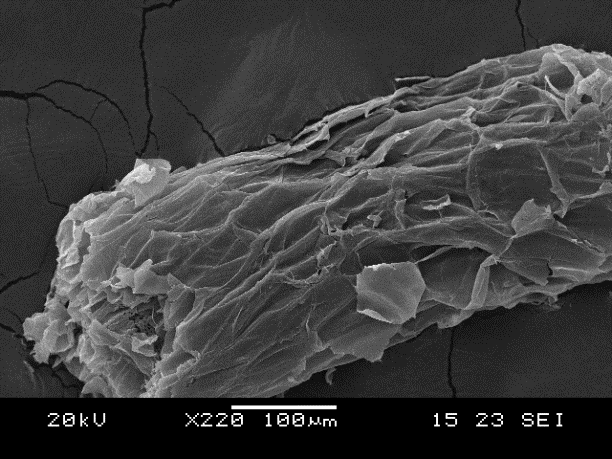

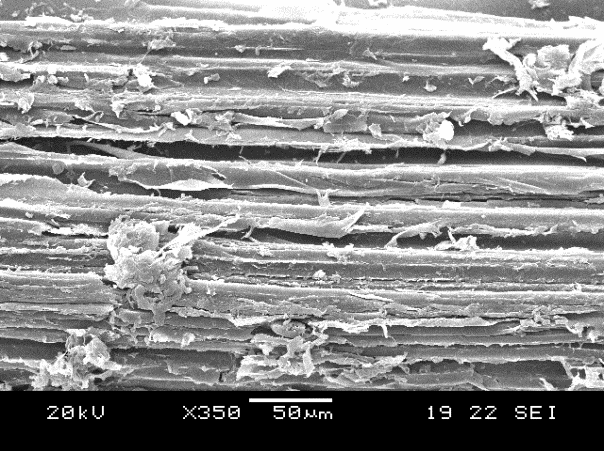

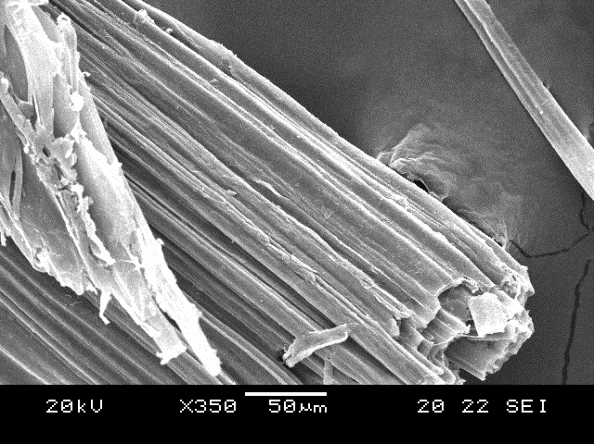

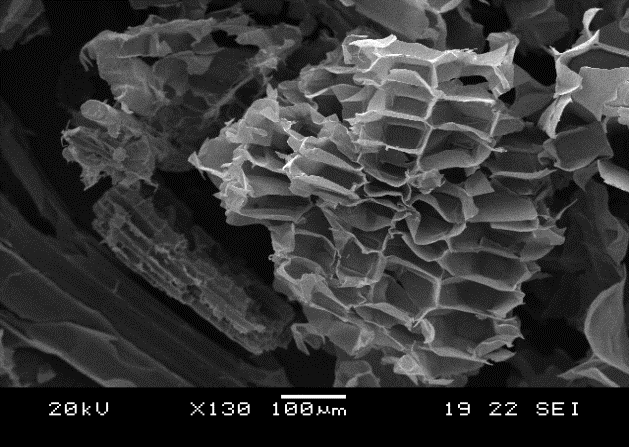


**Figure S4.** Scanning electron micrographs of longitudinal view of, **a** pith bagasse before pretreatment, **b** pith bagasse after pretreatment, **c** long fiber bagasse before pretreatment and, **d**  long fiber bagasse after pretreatment. Treatments were conducted in [TEA][HSO_4_] containing 20 wt% water and solids loading of 10 wt% at 120°C for 4 h.
