# Supplementary material for: Pretreatment of South African sugarcane bagasse using a low-cost protic ionic liquid: a comparison of whole, depithed, fibrous and pith bagasse fractions
Source: Biotechnol Biofuels. 2018 Sep 11;11:247. doi: 10.1186/s13068-018-1247-0 (PMC6131805; doi:10.1186/s13068-018-1247-0)
Supplement: Supplementary file 2 — Additional file 2: Table S1. Chemical composition analysis of untreated South African sugarcane bagasse preparations used in this study. Table S2. Pretreatment outputs. [file 13068_2018_1247_MOESM2_ESM.docx]

**Additional file 2.**

**Table S1.** Chemical composition analysis of untreated South African sugarcane bagasse preparations used in this study.

| **Component / %** | **Whole bagasse (WB)** | **Depithed bagasse (DB)** | **Long fiber bagasse (LFB)** | **Short fiber bagasse (SFB)** | **Pith bagasse (PB)** |
| --- | --- | --- | --- | --- | --- |
| Ethanol-water extractives | 5.6 ± 0.3 | 2.6 ±0.3 | 8.7 ± 0.5 | 10.7 ± 1.9 | 6.7 ± 1.1 |
| Holocellulose (cellulose + hemicelluloses) | 70.7 ± 0.1 | 72.6 ± 1.1 | 68.1 ± 0.4 | 64.8 ± 0.9 | 67.6 ± 0.7 |
| Glucan | 42.3 ± 0.1 | 43.5 ± 0.9 | 42.7 ± 0.2 | 38.1 ± 0.9 | 42.9 ± 0.3 |
| Hemicelluloses (arabinose and xylose) | 28.4 ± 0.1 | 29.1 ± 2.0 | 25.4 ± 0.4 | 26.7 ± 0.3 | 24.7 ± 0.4 |
| Klason lignin | 18.1 ± 0.2 | 19.4 ± 0.8 | 17.8 ± 0.1 | 18.8 ± 0.4 | 18.5 ± 0.5 |
| Acid-soluble lignin | 4.5 ± 0.2 | 4.7 ± 0.2 | 5.0 ± 0.6 | 4.5 ± 0.4 | 5.8 ± 0.1 |
| Total lignin^a^ | 22.6 ± 0.4 | 24.1 ± 0.9 | 22.8 ± 0.7 | 23.3 ± 0.8 | 24.3 ± 0.6 |
| Ash | 1.2 ± 0.3 | 0.7 ± 0.2 | 0.4 ± 0.1 | 1.2 ± 0.1 | 1.5 ± 0.0 |
| Holocellulose:lignin ratio | 3.1 ± 0.1 | 3.0 ± 0.2 | 3.0 ± 0.1 | 2.8 ± 0.1 | 2.8 ± 0.2 |

**Table S2.**

|  | **Whole bagasse (WB)** | **Depithed bagasse (DB)** | **Long fiber bagasse (LFB)** | **Short fiber bagasse (SFB)** | **Pith bagasse (PB)** |
| --- | --- | --- | --- | --- | --- |
| Lignin content in native biomass (wt%) | 22.6 ± 0.4 | 23.7 ± 0.9 | 22.8 ± 0.9 | 23.3 ± 0.8 | 24.3 ± 1.1 |
| Pulp yield (wt%) | 58.5 ± 1.2 | 58.6 ± 0.5 | 43.2 ± 0.3 | 44.6 ± 1.2 | 39.3 ± 0.2 |
| Lignin yield (wt% relative to biomass feedstock) | 11.3 ± 1.2 | 10.6 ± 0.8 | 20.4 ± 2.9 | 16.7 ± 3.1 | 17.2 ± 3.1 |
| Lignin yield (wt% relative to lignin in feedstock) | 49.9 ± 5.1 | 44.7 ± 3.5 | 89.0 ± 12.8 | 72.7 ± 13.3 | 71.7 ± 13.0 |
| Lignin content in pulp (wt%) | 5.6 ± 0.3 | 6.0 ± 0.2 | 2.2 ± 0.0 | 4.2 ± 0.0 | 3.5 ± 0.0 |
| Lignin recovered in pulp (wt% relative to lignin in biomass) | 24.8 ± 1.8 | 25.3 ± 1.8 | 9.6 ± 0.4 | 18.0 ± 0.7 | 14.4 ± 0.7 |
| Delignification (wt%) | 74.9 ± 4.1 | 74.8 ± 2.1 | 90.3 ± 0.5 | 82.2 ± 0.9 | 85.4 ± 0.7 |
| Lignin mass balance* (wt%) | 75.0 ± 9.2 | 69.9 ± 5.6 | 98.7 ± 13.3 | 90.5 ± 14.2 | 86.4 ± 13.8 |
| Ash content in native biomass (wt%) | 1.7 ± 0.3 | 0.7 ± 0.2 | 0.3 ± 0.1 | 1.2 ± 0.1 | 1.4 ± 0.0 |
| Ash recovered in pulp (wt%) | 59 | 59 | 43 | 45 | 39 |
